# Hospitals of To-Day

**Published:** 1911-05-06

**Authors:** 


### HOSPITALS OF TO-DAY.

### IV.—THE POSITION AND NEEDS OF ST. MARY'S HOSPITAL.

The past ten years have brought about a peculiar state of affairs at St. Mary's Hospital which requires a little study for its elucidation. On the one hand the Clarence Wing has been opened, and that section of it which is devoted to the work of Sir Almroth Wright has brought a wide reputation to the hospital, and on the other a continual diminution in the annual sum derived from legacies has forced the hospital to trench on its reserve of invested funds, and has had the effect of postponing part of that work which the completion of the Clarence Wing has led the public to suppose completely accomplished.

This contradictory state of affairs cannot better be illustrated than by a critical survey of the out-patient and casualty departments. The out-patient department, which is on a sub-level, is up to date and equipped for its work; the casualty department and receiving rooms have remained unaltered since 1851, when this wing of the hospital was built. Yet during that period the work which is here dealt with has increased enormously, and 25,000 cases a year are treated to-day in an area that was intended for 4,000 only. It is plain that some curious combination of circumstance must have led to this one-sided development, for even a sciolist knows that out-patient and casualty departments are interdependent and are usually designed or rebuilt at the same time. Why, then, when the out-patient department was modernised were the casualty and receiving rooms left severely alone? Plans for their reconstruction have been prepared for a considerable time, and would have been acted upon had not the stream of charity which caused improvements to be made elsewhere suddenly dried up at the very moment when the remodelling of the casualty department was about to be undertaken. Public support of charities, if indicated on paper, would exhibit a series of catenary curves; a declension began just when this work was to be started.

### The Casualty Department, as It Is.

The casualty department has not been improved, for the determination of the Board to have the necessary alterations has led them wisely to refrain from tinkering with a series of rooms that, it is no exaggeration to say, no tinkering could make adequate. The rooms are small and consequently liable to be crowded, and patients often have to wait a very long time for their turns. This smallness also means that they must wait in discomfort, for the accommodation is limited and can be eked out only by screens. The sexes are separated under difficulties, and the waiting patients may have to beguile their expectancy with the sights and sounds from which they should be free. For instance, there is no accommodation for patients recovering from the effects of an anæsthetic after minor operations, and if an infectious case is discovered no proper segregation can be enforced. No one has known and regretted this state of affairs more than the hospital's Board, and one wonders whether to admire more the splendid work that has been done under these conditions, or to regret the conditions that still accompany it.

The Board has decided, however, to tolerate these conditions no longer, and the state of affairs just described, fortunately for the patients, will soon be ancient history. The work of reconstruction is to be begun during the summer vacation, and an appeal is now being issued. The state of affairs which has led to this appeal has been dealt with. It remains to say something about the details of reconstruction. When the Clarence Wing was opened the board-room and the secretarial offices were transferred to it, leaving their old quarters free to be adapted to another purpose. These quarters, particularly the old board-room, which is of the fine dimensions associated in mid-Victorian days with the responsibilities of administrative office, will be the nucleus of a new casualty department. At present one end of the ward-room is used as a dining-room for the resident medical officers. This will be made theirs permanently. The remainder of the room will form the extreme end of the casualty department.

### As It Will Be.

Roughly speaking, the whole of the ground floor on the right of the entrance to the hospital in Cambridge Place will be gutted. It is impossible to describe further the structural rearrangements without a plan, as the present arrangements themselves are the result of an alteration caused by dividing the great height of the ground floor rooms into two by an added ceiling, which produces a difference in height between the ground floor as seen from the foot of the great stair, and the ground floor as seen from the corridor a few feet nearer Cambridge Place. The ingenious plan of rearrangement adopted will cost £7,500, and there can be no doubt at all that an equal sum of money has never done more for the comfort of patients than this will do when its effects are complete. Moreover, no additional sum for maintenance will be required when the alterations have been made. This is an appeal for the patients if ever there was one.

During the past ten years, which have made the inadequacy of the casualty departments so lamentably clear, St. Mary's finances have also been subjected to expedients. Nine lean years have proved a terrible strain, and have resulted in a total loss of over £39,000. This loss has been met by the sale of £34,500 stock and by the long hoped for advent of a good year (1910), the whole surplus of which was wiped out in a final attempt to pay off the whole. This has been done, with the exception of £1,000, and the problem before the hospital is to obtain a capital sum with which to make good the amount taken out of its invested funds. Till that is done St. Mary's will only be recovering lost ground, and when that is done it will be time enough to talk of further improvement.

